# The role of transcriptional factor p63 in regulation of epithelial barrier and ciliogenesis of human nasal epithelial cells

**DOI:** 10.1038/s41598-017-11481-w

**Published:** 2017-09-07

**Authors:** Yakuto Kaneko, Takayuki Kohno, Takuya Kakuki, Ken-ichi Takano, Noriko Ogasawara, Ryo Miyata, Shin Kikuchi, Takumi Konno, Tsuyoshi Ohkuni, Ryoto Yajima, Akito Kakiuchi, Shin-ichi Yokota, Tetsuo Himi, Takashi Kojima

**Affiliations:** 1 Department of Otolaryngology, Sapporo Medical University School of Medicine, Sapporo, 060-8556 Japan; 2 Department of Microbiology, Sapporo Medical University School of Medicine, Sapporo, 060-8556 Japan; 3 Department of Anatomy, Sapporo Medical University School of Medicine, Sapporo, 060-8556 Japan; 4 Department of Cell Science, Research Institute for Frontier Medicine, Sapporo Medical University School of Medicine, Sapporo, 060-8556 Japan

## Abstract

Disruption of nasal epithelial tight junctions (TJs) and ciliary dysfunction are found in patients with chronic rhinosinusitis (CRS) and nasal polyps (NPs), along with an increase of p63-positive basal cells and histone deacetylase (HDAC) activity. To investigate these mechanisms, primary cultures of HNECs transfected with human telomerase reverse transcriptase (hTERT-HNECs) were transfected with siRNAs of TAp63 and ΔNp63, treated with the NF-kB inhibitor curucumin and inhibitors of HDACs, and infected with respiratory syncytial virus (RSV). In TERT-HNECs, knockdown of p63 by siRNAs of TAp63 and ΔNp63, induced claudin-1 and -4 with Sp1 activity and enhanced barrier and fence functions. The knockdown of p63 enhanced the number of microvilli with the presence of cilia-like structures. Treatment with curcumin and inhibitors of HDACs, or infection with RSV prevented expression of p63 with an increase of claudin-4 and the number of microvilli. The knockdown or downregulation of p63 inhibited phospho-p38MAPK, and the p38MAPK inhibitor downregulated p63 and upregulated the barrier function. Thus, epithelial barrier and ciliogenesis of nasal epithelium are regulated in a p63-negative manner in normal and upper airway diseases. Understanding of the regulation of p63/p38 MAPK/NF-κB may be important in the therapy for airway allergy and its drug delivery system.

## Introduction

The airway epithelium of the human nasal mucosa acts as a physical barrier that protects against inhaled substances and pathogens via tight junctions (TJs)^1–3^.

TJs, the most apically located of the intercellular junctional complexes, have epithelial barrier and fence functions^4–6^. TJs are modulated by various intracellular signaling pathways to affect the epithelial barrier function in response to some cytokines, growth factors, and hormones^7, 8^^,^. TJs are formed by not only the integral membrane proteins claudins (CLDNs), occludin (OCLN), and junctional adhesion molecules (JAMs), but also many peripheral membrane proteins^6, 9^. Recently, tricellulin (TRIC) and lipolysis-stimulated lipoprotein receptor (LSR) were identified at tricellular contacts where there are three epithelial cells and shown to have a barrier function^10^.

The CLDN family, consisting of 27 members, is solely responsible for forming tight junction strands and shows tissue- and cell-specific expression of individual members^11^. Several lines of evidence point to claudins as the basis for the selective size, charge, and conductance properties of the paracellular pathway^12^. The C-terminal fragment of Clostridium perfringens enterotoxin (C-CPE) binds the claudins and disrupts the epithelial barrier without a cytotoxic effect in nasal epithelium^13, 14^.

It is known that there are transcriptional regulators of claudins in epithelial TJs^15^. Sp1, cdx-2, FoxO1, ELF3 and HNF4α are the transcriptional factors of various claudins^15^. In addition, the promoters of CLDN-1 and CLDN-4 are controlled by epigenetic modifications of the Sp1-containing critical promoter region^16^. EGF activates a MEK/ERK pathway and increases Sp1 expression, resulting in an elevation of CLDN-4 expression in MDCK cells^17^. Sodium butyrate (NaB) enhances the intestinal barrier function through an increase of CLDN-1 expression via Sp1^18^.

In the human nasal mucosa in which many cilia are observed on the surface, expression of occludin, JAM-A, ZO-1, ZO-2, CLDN-1, -4, -7, -8, -12, -13, -14, tricellulin and LSR is detected^3^. In the human nasal epithelium, occludin, JAM-A and ZO-1 are found in the uppermost layer and claudin-1 in the uppermost and basal layers, whereas CLDN-4 and CLDN-7 are observed throughout the epithelium^3^.

A defective epithelial barrier with decreased expression of TJ proteins is found in patients with chronic rhinosinusitis (CRS) and nasal polyps (NPs)^19^. The nasal epithelial CLDN-4 is markedly upregulated by TGF-β, which is closely related to NPs, CRS and human respiratory syncytial virus (RSV)-infection^6, 20, 21^.

Transcriptional factor p63, which is a member of the p53 family and has two distinct isoforms, TAp63 and ΔNp63, plays an important role in the proliferation and differentiation of various epithelial basal cells^22^. It is known that p63 is upstream of IKKα in epidermal development^22^. p63 is also one of the regulators of various cell–matrix and cell–cell adhesion complexes in the epidermis^23^. It contributes to the formation and maintenance of differentiated pseudostratified bronchial epithelium^24^. ΔNp63 plays a critical role in epithelial stratification and in skin stem cell renewal^25^. Loss of ΔNp63 significantly reduces epithelial proliferation and increases E-cadherin expression in human airway epithelial cells^26^.

An increase in p63-positive cells is observed in the epithelium of NPs and the expression of p63 in multiple cell layers is an important pathologic phenomenon in the epithelial remodeling seen in NPs^27, 28^. RSV infects p63^+^ airway basal cells in air-liquid interface cultures of human bronchial epithelial cells and influences p63 expression and differentiation^29^.

On the other hand, the ciliary dysfunction also occurs in chronic rhinosinsusitis^30^. Transcriptional factor Myb (+) cells are increased in chronic airways disease^31^. A p63(−) Myb(+) population arising from self-renewing p63 (+) Krt5 (+) epithelial progenitors in airway epithelial cells represents a distinct intermediate stage of differentiation towards ciliated cells under the influence of specific regulatory factors, including Notch and FOXJ1^31^. It is possible that the epithelial barrier created by TJ proteins and cilia formation in the upper airway are regulated via p63.

Histone deacetylase (HDACs) are a class of enzymes that remove acetyl groups from the lysine residues of target proteins, thereby promoting chromatin condensation and reduced transcription^32^. Eighteen mammalian HDACs have been identified to date and they are divided into 4 classes: class I HDACs (HDACs 1, 2, 3, and 8), class II HDACs (HDACs 4, 5, 6, 7, 9, and 10), class IV (HDAC 11), and class III (sirtuin family: SIRT1-SIRT7)^33^. Class II HDACs are further divided into two subgroups: Class IIa (4, 5, 7, and 9) and IIb (6 and 10).

Expression of HDAC1 and HDAC9 is high in bronchial epithelial cells (HBECs) from asthmatic patients and the inhibition of HDAC activity reconstitutes a defective barrier by increasing TJ expression^34^. HDAC is an important epigenetic regulator in RSV-induced lung inflammation and treatment with inhibitors of HDAC inhibits RSV replication and decreases RSV-induced airway inflammation and oxidative stress^35^. HDAC inhibitors induce cell death, the cell cycle, senescence, differentiation, autophagy and tumor immunogenicity^36^. The HDAC inhibitor sodium butyrate significantly upregulates the protein levels of cingulin, ZO-1, and ZO-2 in Rat-1 fibroblasts, cingulin in COS-7 cells, and cingulin and occludin in HeLa cells^37^.

In the present study, p63, ΔNp63, HDAC1 and HDAC6 were upregulated and CLDN-1 and -4 were downregulated in the epithelium of sinusitis and NPs. In HNECs, knockdown of p63 and ΔNp63 induced expression of CLDN-1 and -4, enhanced barrier and fence functions, and increased the number of microvilli on the cell surface. Inhibitors of NF-κB, HDACs and p38 MPAK, and RSV infection, prevented p63 expression and induced TJ proteins, p63-negative regulation of the epithelial barrier and ciliogenesis of the nasal epithelium.

## Methods

### Ethics statement

The protocol for human study was reviewed and approved by the ethics committee of the Sapporo Medical University School of Medicine. Written informed consent was obtained from each patient who participated in the investigation. All experiments were carried out in accordance with the approved guidelines and with the Declaration of Helsinki.

### Antibodies and reagents

A mouse monoclonal anti-p63 (DAK-p63) antibody was obtained from Dako (Tokyo, Japan). A rabbit polyclonal anti-p63 antibody was from Abcam (Cambridge, MA, USA). A rabbit polyclonal anti-p40 (ΔNp63) antibody was from NICHIREI BIOSCIENCES INC. (Tokyo, Japan). A rabbit polyclonal anti-ΔNp63 antibody was from BioLegend (Tokyo, Japan). Mouse monoclonal anti-cytokeratin5 (CK5) and anti-cytokeratin-7 (CK7) antibodies were from Sigma Aldrich. Rabbit polyclonal anti-CLDN-1, -4, and -7, anti-occludin, anti-tricellulin and mouse monoclonal anti-CLDN-4 (3E2C1) antibodies were from Zymed Laboratories (San Francisco, CA). A rabbit polyclonal anti-LSR antibody was from Novus Biologicals (Littleton, CO, USA). Mouse monoclonal anti-HDAC1 (10E2) and rabbit polyclonal anti-HDAC6, anti-phospho-NFκB, anti-NFκB, anti-phospho-p38 MAPK and anti-p38 MAPK antibodies were from Cell Signaling Technology (Danvers, MA, USA). Mouse monoclonal anti-acetylated tubulin (T7451), anti-γ-tubulin (GTU-88) and rabbit polyclonal anti-actin antibodies were from Sigma-Aldrich Inc. (St. Louis, MO). Alexa 488 (green)-conjugated anti-rabbit IgG and Alexa 594 (red)-conjugated anti-mouse IgG antibodies were from Molecular Probes, Inc. (Eugene, OR). A NF-kB inhibitor curcumin was purchased from Cayman Chemical Corporation (Ann Arbor, MI). A p38 MAPK inhibitor SB203580 was purchased from Calbiochem-Novabiochem Corporation (San Diego, CA). Trichostatin A (TSA) was from Sigma-Aldrich (St. Louis, MO, USA). Inhibitors of HDAC1 and HDAC6 were from Santa Cruz Biotechnology (Dallas, TX, USA). HRP-conjugated polyclonal goat anti-rabbit IgG was from Dako A/S (Glostrup, Denmark). The ECL Western blotting system was from GE Healthcare UK, Ltd. (Buckinghamshire, UK).

### GeneChip analysis

Microarray slides were scanned using a 3D-GENE human 25k. (TORAY, Tokyo, Japan) and microarray images were automatically analyzed using AROS^TM^, version 4.0 (Operon Biotechnologies, Tokyo, Japan).

### Immunohistochemical analysis

Human nasal tissues were obtained from each 10 patients with hypertrophic rhinitis or chronic sinusitis who underwent inferior turbinectomy at Sapporo Medical University, the Sapporo Hospital of Hokkaido Railway Company, or the KKR Sapporo Medical Center Tonan Hospital. Informed consent was obtained from all patients and this study was approved by the ethics committees of the above institutions.

The tissues were embedded in paraffin after fixation with 10% formalin in PBS. Briefly, 5-μm-thick sections were dewaxed in xylene, rehydrated in ethanol, and heated with Vision BioSystems Bond Max using ER2 solution (Leica) in an autoclave for antigen retrieval. Endogenous peroxidase was blocked by incubation with 3% hydrogen peroxide in methanol for 10 min. The tissue sections were then washed twice with Tris-buffered saline (TBS) and preblocked with Block Ace for 1 h. After washing with TBS, the sections were incubated with anti-p63, anti-ΔNp63, anti-claudin-1, anti-claudin-4, anti-claudin-7, anti-HDAC1 and anti-HDAC6 antibodies (1:400) for 1 h. The sections were then washed three times in TBS and incubated with Vision BioSystems Bond Polymer Refine Detection kit DS9800. After three washes in TBS, a diamino-benzidine tetrahydrochloride working solution was applied. Finally, the sections were counterstained with hematoxylin.

### Cell culture and treatments

The cultured HNECs were derived from mucosal tissues of patients with hypertrophic rhinitis or chronic sinusitis who underwent inferior turbinectomy at Sapporo Medical University, the Sapporo Hospital of Hokkaido Railway Company, or the KKR Sapporo Medical Center Tonan Hospital. Informed consent was obtained from all patients and this study was approved by the ethics committees of the above institutions.

The methods for primary culture of human nasal epithelial cells were as reported previously (Koizumi *et al*., 2008). Some primary cultured HNECs were transfected with the catalytic component of telomerase, the human catalytic subunit of the telomerase reverse transcriptase (hTERT) gene, as described previously (Kurose *et al*., 2007). The cells were plated on 35-mm or 60-mm culture dishes (Corning Glass Works, Corning, NY), which were coated with rat tail collagen (500 μg of dried tendon/ml 0.1% acetic acid). The cells were cultured in serum-free bronchial epithelial cell basal medium (BEBM, Lonza Walkersville, Inc.; Walkersville, MD) supplemented with bovine pituitary extract (1% v/v), 5 μg/ml insulin, 0.5 μg/ml hydrocortisone, 50 μg/ml gentamycin, 50 μg/ml amphotericin B, 0.1 ng/ml retinoic acid, 10 μg/ml transferrin, 6.5 μg/ml triiodothyronine, 0.5 μg/ml epinephrine, 0.5 ng/ml epidermal growth factor (Lonza Walkersville, Inc.), 100 U/ml penicillin and 100 μg/ml streptomycin (Sigma-Aldrich) and incubated in a humidified, 5% CO_2_:95% air incubator at 37 °C. In this experiment, 2nd and 3rd passaged cells were used.

Some cells cultured with or without FBS, were treated with 1 and 5 μM curcumin, 1 and 10 μM TSA, 1 and 10 μM inhibitors of HDAC1 and HDAC6 or 10 μM SB203580.

### siRNA experiment

For knockdown of human TAp63 and human ΔNp63, Stealth^TM^ Select RNAi against the genes was synthesized by Invitrogen (Carlsbad, CA). The sequences were as follows: siRNA of TAp63 (sense: 5′-GGAAUGACUUCAACUUUGA-3′; antisense: 5′-UCAAAGUUGAAGUCAUUCC-3′), siRNA of ΔNp63 (sense: 5′-ACAAUGCCCAGACUCAAUU-3′; antisense: 5′-AAUUGAGUCUGGGCAUUGU-3′). hTERT-HNECs cultured without FBS at 24 h after plating were transfected with 100 nM siRNAs using Lipofectamine^TM^ RNAiMAX Reagent (Invitrogen) for 48 h. A scrambled siRNA sequence (BLOCK-iT Alexa Fluor fluorescent, Invitrogen) was employed as control siRNA.

### RSV experiment

RSV was grown in the human laryngeal carcinoma cell line HEp-2. For infection, HNECs at 80% confluence were adsorbed at an RSV multiplicity of infection (MOI) of 1 for 60 min at 37 °C. After adsorption, the viral solutions were removed and the cells were rinsed twice with growth medium and incubated. The virus titers in the supernatant were determined by a plaque-forming assay with HEp-2 cells. Expression of RSV mRNA was confirmed by reverse transcription-PCR (RT-PCR). Some cells cultured without FBS were infected with RSV for 24 h^21^.

### Luciferase reporter assay

Cells were seeded on 12-well plates in triplicate and allowed to grow overnight to reach 50–70% confluence. The cells were cotransfected with SP1 reporter and siRNAs of p63 and ΔNp63. After 48 h of transfection, Luciferase activity was measured using the Dual Luciferase Reporter Assay System kit (Promega) in a TECAN microplate reader (Infinite M1000 Pro, Tecan Japan Co., Ltd., Kawasaki, Japan). Luciferase activity was normalized to *R*. *reniformis* luciferase activity and plotted as mean ± SD from three independent experiments.

### Western blot analysis

The hTERT-transfected HNECs were scraped from a 60 mm dish containing 300 μl of buffer (1 mM NaHCO3 and 2 mM phenylmethylsulfonyl fluoride), collected in microcentrifuge tubes, and then sonicated for 10 s. The protein concentrations of the samples were determined using a BCA protein assay reagent kit (Pierce Chemical Co.; Rockford, IL). Aliquots of 15 μl of protein/lane for each sample were separated by electrophoresis in 5–20% SDS polyacrylamide gels (Wako, Osaka, Japan), and electrophoretically transferred to a nitrocellulose membrane (Immobilon; Millipore Co.; Bedford, UK). The membrane was saturated for 30 min at room temperature with blocking buffer (25 mM Tris, pH 8.0, 125 mM NaCl, 0.1% Tween 20, and 4% skim milk) and incubated with anti-CK5, anti-CK7, anti-p63, anti-ΔNp63, anti-anti-RSV-G protein^21^, anti-occludin, anti-CLDN-1, -4, -7, anti-LSR, anti-tricellulin, anti-Ac-tubulin, anti-γ-tubulin, anti-phospho-NFκB, anti-NFκB, anti-phospho-p38 MAPK and anti-p38 MAPK antibodies (1:1000) at room temperature for 1 h. Then it was incubated with HRP-conjugated anti-mouse and anti-rabbit IgG antibodies at room temperature for 1 h. The immunoreactive bands were detected using an ECL Western blotting system.

### Immunocytochemistry

hTERT-transfected HNECs grown in 35mm glass-coated wells (Iwaki, Chiba, Japan), were fixed with cold acetone and ethanol (1:1) at –20 °C for 10 min. After rinsing in PBS, the cells were incubated with anti-CK5, anti-CK7, anti-p63, ΔNp63, anti-anti-RSV-G protein (Masaki *et al*., 2011), and anti-occludin, anti-CLDN-4, anti-LSR, anti-tricellulin, anti-Ac-tubulin and anti-γ-tubulin antibodies (1:100) overnight at 4 °C. Alexa Fluor 488 (green)-conjugated anti-rabbit IgG and Alexa Fluor 592 (red)-conjugated anti-mouse IgG (Invitrogen) were used as secondary antibodies. The specimens were examined and photographed with an Olympus IX 71 inverted microscope (Olympus Co.; Tokyo, Japan) and a confocal laser scanning microscope (LSM510; Carl Zeiss, Jena, Germany).

### Scanning electron microscopy (SEM)

Cells grown on coated coverslips were fixed with 2.5% glutaraldehyde/0.1 M PBS (pH 7.3) overnight at 4 °C. After several rinses with PBS, the cells were postfixed in 1% osmium tetroxide at 4 °C for 3 h and then rinsed with distilled water, dehydrated in a graded ethanol series, and freeze-dried. The specimens were sputter-coated with platinum and observed with a scanning electron microscope (S-4300, Hitachi; Tokyo, Japan) operating at 10 kV.

### Measurement of transepithelial electrical resistance (TEER)

hTERT-transfected HNECs were cultured to confluence in the inner chambers of 12-mm Transwell inserts with 0.4-µm pore-size filters (Corning Life Sciences). TER was measured using an EVOM voltameter with an ENDOHM-12 (World Precision Instruments, Sarasota, FL) on a heating plate (Fine, Tokyo, Japan) adjusted to 37 °C. The values were expressed in standard units of ohms per square centimeter and presented as the mean ± S.D. For calculation, the resistance of blank filters was subtracted from that of filters covered with cells.

### Diffusion of BODIPHY-sphingomyelin

For measurement of the tight junctional fence function, we used diffusion of BODIPY-sphingomyelin with some modification^38^. Sphingomyelin/BSA complexes (5 nM) were prepared in P buffer (10 nM HEPES, pH 7.4, 1 mM sodium pyruvate, 10 mM glucose, 3 mM CaCl_2_, and 145 mM NaCl) using BODIPY-FL-sphingomyelin (Molecular Probes) and defatted BSA. Cells plated on glass-bottom microwell plates (Mat Tek Corp., Ashland, MA) were loaded with BODIPY-sphingomyelin/BSA complex for 1 min on ice, after which they were rinsed with cold DMEM and mounted in DMEM on a glass slide. The samples were analyzed by confocal laser scanning microscopy (LSM5; Carl Zeiss, Jena, Germany). All pictures shown were generated within the first 5 min of analysis.

### Data analysis

Signals were quantified using Scion Image Beta 4.02 Win (Scion Co.; Frederick, MA). Each set of results shown is representative of at least three separate experiments. Results are given as means ± SEM. Differences between groups were tested by ANOVA followed by a posthoc test and an unpaired two-tailed Student’s t test and considered to be significant when p < 0.05.

## Results

### Expression and localization patterns of p63, ΔNp63 and CLDN-1, -4, and -7 in the nasal epithelium of sinusitis and polyps

**Figure 1:**
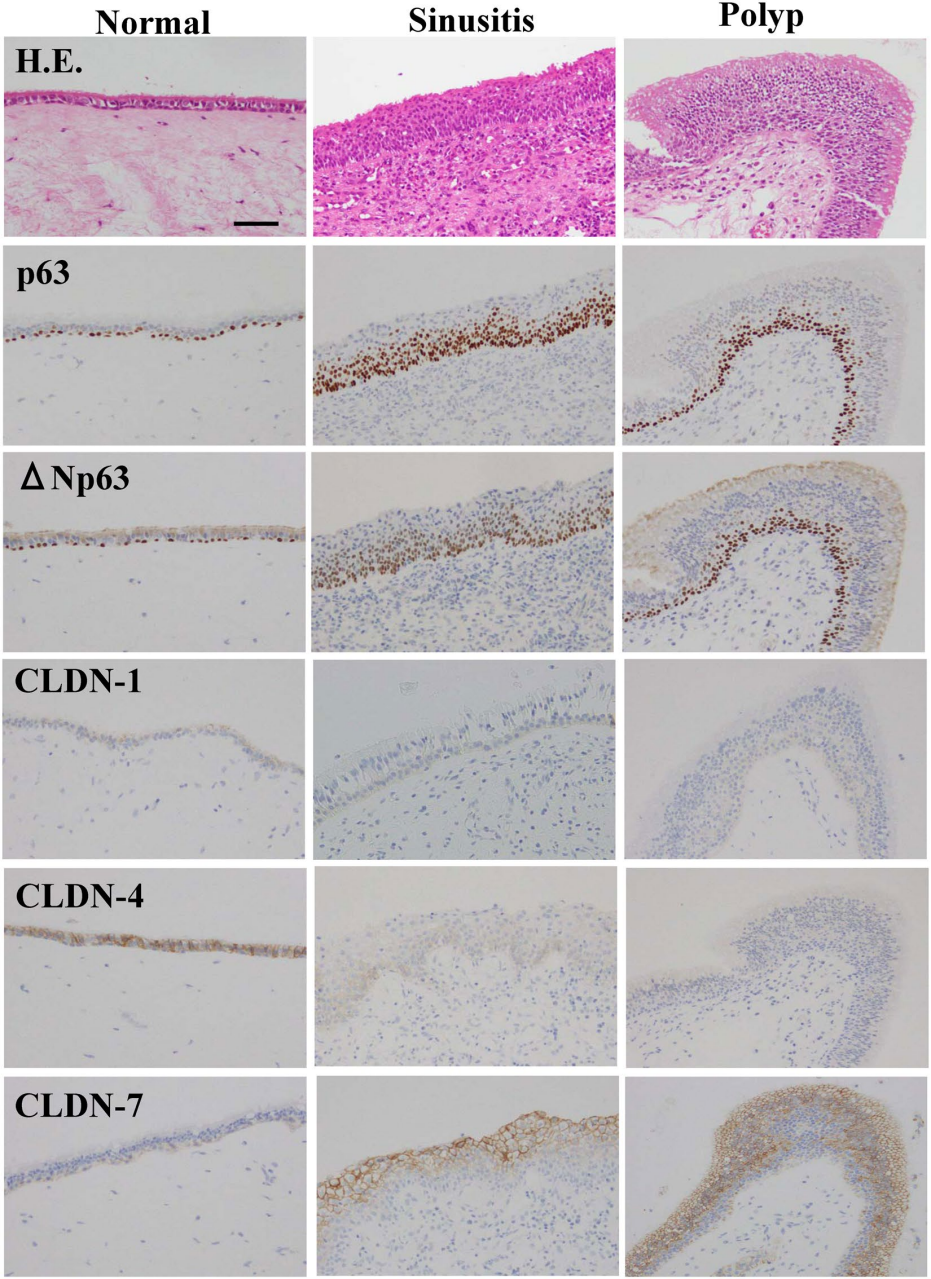
Images of H.E. and immunohistochemical staining of p63, p40 (ΔNp63), claudin-1, -4 and -7 in normal nasal mucosal tissues and those from patients with, sinusitis and polyps. Bar: 50 μm.

We performed immunohistochemical analysis for p63, ΔNp63 and CLDN-1, -4, -7 in normal, sinusitis and polyp (NP) tissues. In the normal nasal epithelium, p63 and ΔNp63 were positive in the nuclei of the basal cell type, and in the epithelium of sinusitis and NPs, p63 andΔNp63 were upregulated (Fig. 1). CLDN-1 and CLDN-4 were downregulated in the sinusitis and NPs, whereas no change of CLDN-7 was observed in sinusitis or NPs (Fig. 1).

### Upregulation of tight junction proteins by siRNAs of TAp63 and ΔNp63 in HNECs

**Figure 2:**
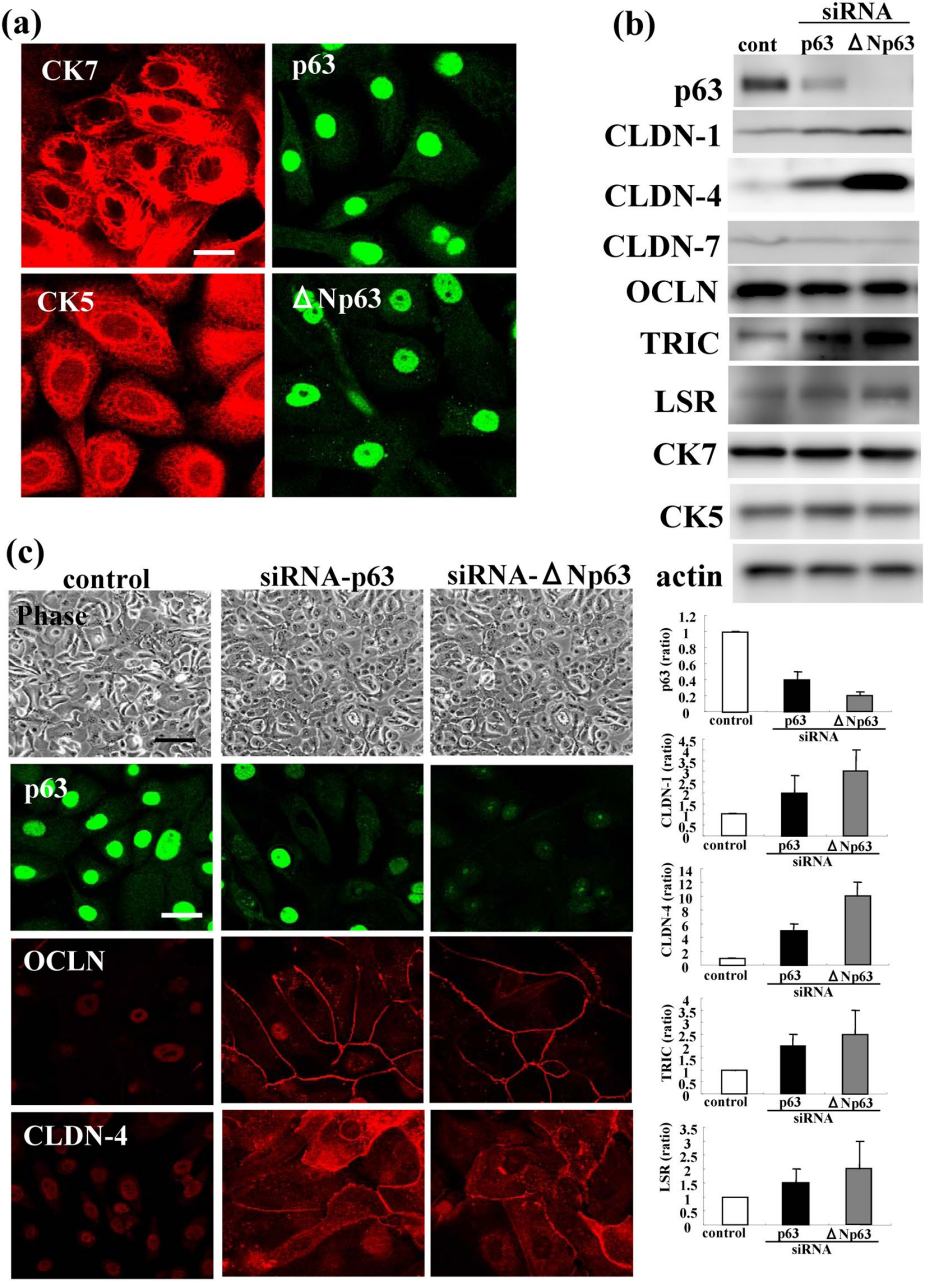
(**a**) Images of immunocytochemical staining for CK5, CK7, p63 and ΔNp63 in hTERT-HNECs. Bar: 20 μm. (**b**) Western blotting for p63, claudin-1, -4, -7, OCLN, TRIC, LSR, CK5 and CK7 in hTERT-HNECs transfected with siRNAs of p63 and ΔNp63. The corresponding expression levels are shown as bar graphs. (**c**) Phase-contrast images and immunocytochemical staining for p63, OCLN and claudin-4 in hTERT-HNECs transfected with siRNAs of p63 and ΔNp63. Bar: 20 μm.

To investigate the mechanisms involved in the regulation of TJs via p63 in HNECs, hTERT-HNECs cultured without FBS, were transfected with siRNAs of TAp63 and ΔNp63. hTERT-HNECs cultured without FBS were detected CK7, CK5, p63 and ΔNp63 (Fig. 2a). In Western blotting, knockdown of p63 by siRNAs of TAp63 and ΔNp63 induced CLDN-1, -4, TRIC and LSR (Fig. 2b). The immunocytochemical results showed that OCLN and CLDN-4 presented at the membranes of the cells, which reduced p63 expression by siRNAs of TAp63 and ΔNp63, whereas OCLN and CLDN-4 were not detected in control cells without FBS (Fig. 2c). Furthermore, tricellular tight junction proteins TRIC and LSR were presented at the membranes by siRNAs of TAp63 and ΔNp63, whereas they were not detected in control cells without FBS (Supplemental Fig. 1).

### Upregulation of Sp1 by siRNAs of TAp63 and ΔNp63 in HNECs

**Figure 3:**
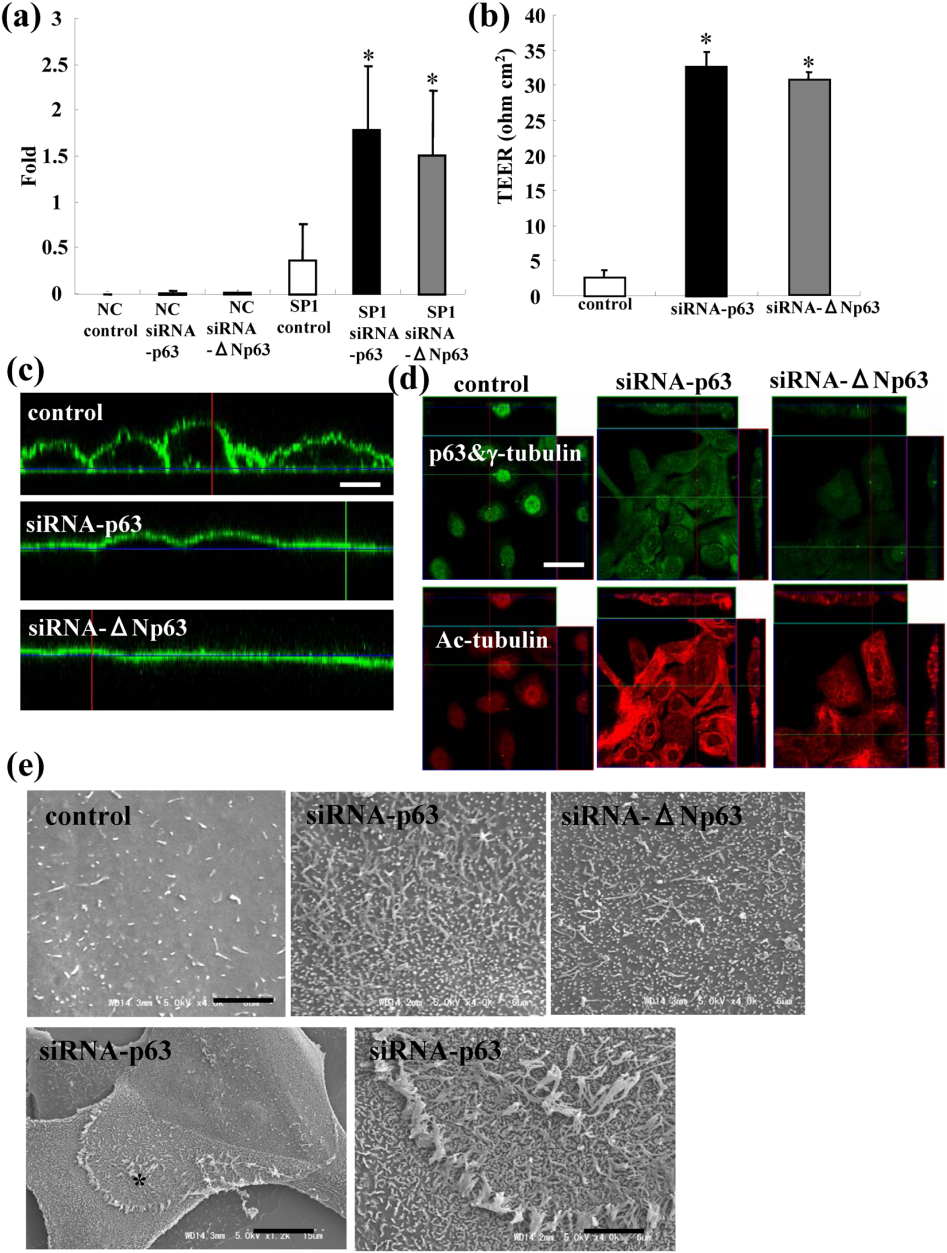
(**a**) SP1 reporter activity values and (**b**) TEER values representing barrier function in hTERT-HNECs transfected with siRNAs of p63 and ΔNp63. The corresponding expression levels are shown as bar graphs. (**c**) Images of diffusion of labeled BODIPY-sphingomyelin indicating fence function, (**d**) immunocytochemical staining for p63, γ-tubulin and Ac-tubulin and (**e**) SEM in hTERT-transfected HNECs transfected with siRNAs of p63 and ΔNp63. White bar: 20 μm, black bar: 3 μm.

To investigate whether Sp1 activity contributed to induction of CLDN-1 and -4 by siRNAs of TAp63 and ΔNp63 in HNECs, we performed luciferase reporter assay for Sp1. Knockdown of p63 by siRNAs of TAp63 and ΔNp63 induced Sp1-luciferase activity (Fig. 3a).

### Upregulation of epithelial barrier and fence functions by siRNAs of TAp63 and ΔNp63 in HNECs

To investigate the epithelial barrier and fence functions affected via p63 in HNECs, we measured the TEER values of hTERT-HNECs transfected with siRNAs of TAp63 and ΔNp63 to determine the barrier function and examined diffusion of BODIPY-sphingomyelin for the fence function. Knockdown of p63 by siRNAs of TAp63 and ΔNp63 induced the epithelial barrier and maintained the fence function, whereas the TEER value was low and the fence function was absent in control cells cultured without FBS (Fig. 3b,c).

### Increase of surface microvilli and presence of cilia-like structures induced by siRNAs of TAp63 and ΔNp63 in HNECs

To investigate the ciliogenesis mediated via p63 in HNECs, hTERT-HNECs transfected with siRNAs of TAp63 and ΔNp63 were examined by immunocytochemistry for Ac-tubulin and by SEM of the surface. Knockdown of p63 by siRNAs of TAp63 and ΔNp63 induced Ac-tubulin expression and enhanced the number of microvilli on the cell surface together with the presence of cilia-like structures. (Fig. 3e).

### Upregulation of tight junction proteins via p63 by NF-κB inhibitor curcumin in HNECs

**Figure 4:**
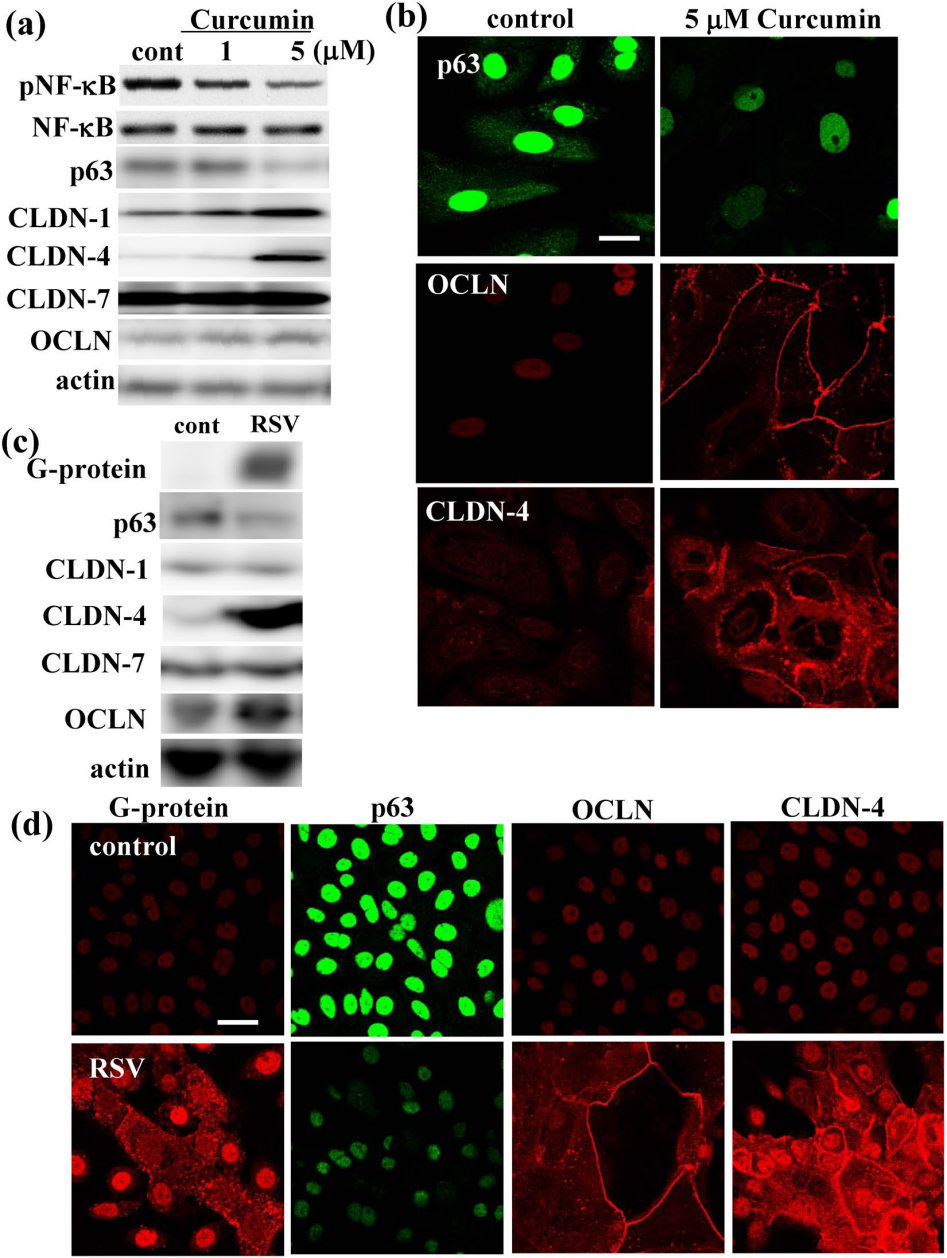
(**a**) Western blotting for phospho-NF-κB, NF-κB, p63, claudin-1, -4, -7 and OCLN and (**b**) images of immunocytochemical staining for p63, OCLN and claudin-4 in hTERT-transfected HNECs treated with 1 or 5 μM curcumin. Bar: 20 μm. (**c**) Western blotting for G-protein, p63, claudin-1, -4, -7 and OCLN and (**d**) Immunocytochemical staining for p63, OCLN and claudin-4 in hTERT-transfected HNECs infected with RSV (MOI = 1). Bar: 20 μm.

p65/NF-κB regulates p63 expression^39^. In Western blotting, treatment with the NF-κB inhibitor curcumin prevented p63 expression and induced expression of CLDN-1, -4 and OCLN in hTERT-HNECs (Fig. 4a, Supplemental Fig. 3A). The immunocytochemical results showed that OCLN and CLDN-4 presented at the membranes of the cells that had reduced p63 due to the treatment with curcumin, whereas OCLN and CLDN-4 were not detected in control cells without FBS (Fig. 4b).

### Upregulation of tight junction proteins via p63 by RSV infection in HNECs

We previously reported that OCLN and CLDN-4 were upregulated via NF-κB by infection with RSV in HNECs^21^. We therefore investigated whether RSV-infection upregulated OCLN and CLDN-4 via p63 in hTERT-HNECs. In Western blotting, RSV infection at MOI 1 for 24 h downregulated p63 and upregulated OCLN and CLDN-4 (Fig. 4c, Supplemental Fig. 3B). The immunocytochemical results showed that OCLN and CLDN-4 presented at the membranes of the cells that were G-protein positive and had reduced p63 due to RSV infection (Fig. 4d).

### Expression patterns of HDAC1 and HDAC6 in the nasal epithelium of sinusitis and polyps

**Figure 5:**
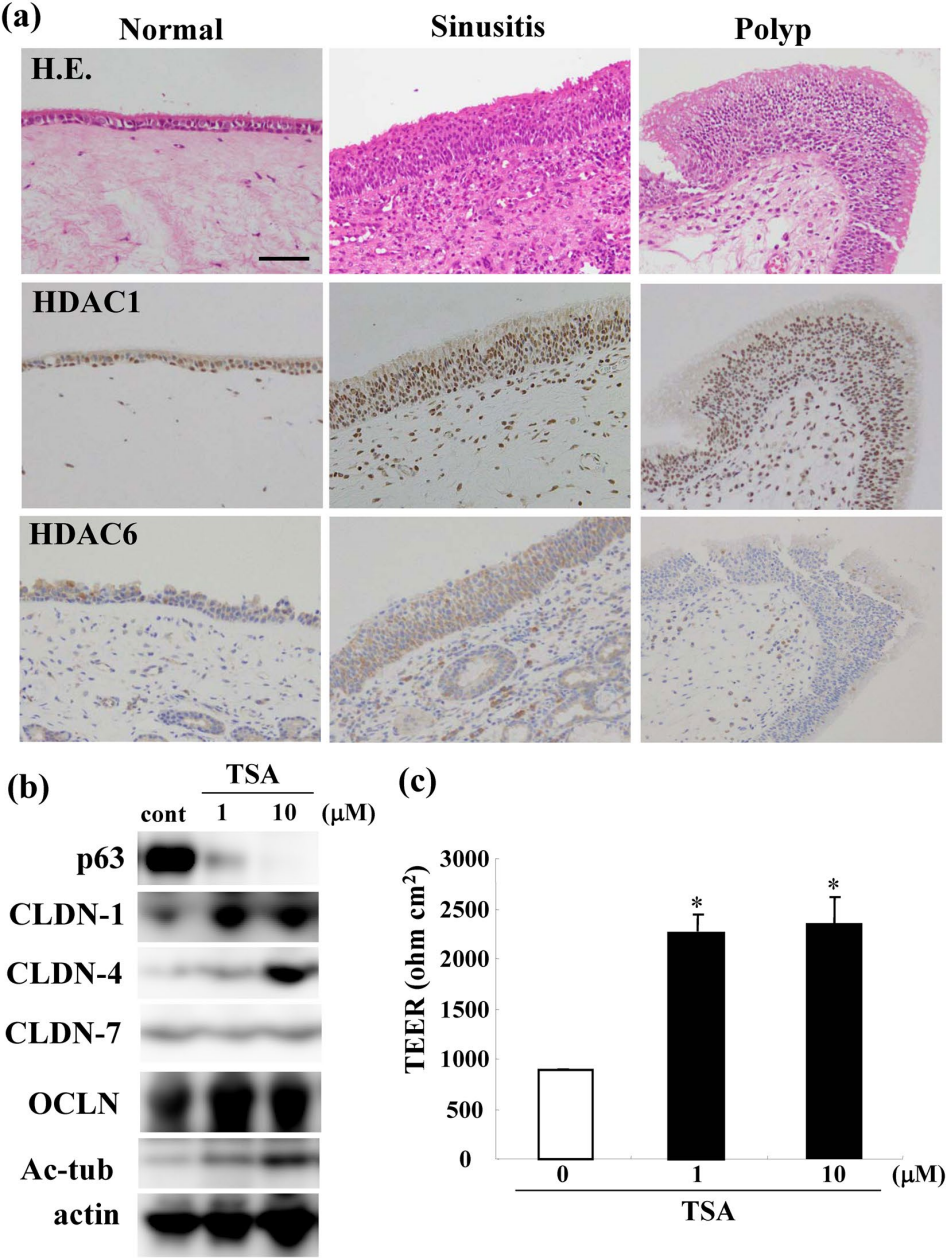
(**a**) Images of H.E. and immunohistochemical staining of HDAC1 and HDAC6 in normal nasal mucosal tissues and those from patients with sinusitis and polyps. Bar: 50 μm. (**b**) Western blotting for p63, claudin-1, -4, -7, OCLN, TRIC, LSR, CK5 and CK7 and (**c**) TEER values representing barrier function in hTERT-HNECs treated with 1 or 10 μM TSA.

We performed immunohistochemical analysis for HDAC1 and HDAC6 in normal, sinusitis and NP tissues. The immunohistochemical results showed that HDAC1 was upregulated in the nasal epithelium of the sinusitis and NPs, and HDAC6 was upregulated in the sinusitis, whereas HDAC1 and HDAC6 were positive in the normal nuclei (Fig. 5a).

### Upregulation of tight junction proteins, barrier function and surface microvilli by the pan-inhibitor of HDACs Tricostatin A (TSA) in HNECs

**Figure 6:**
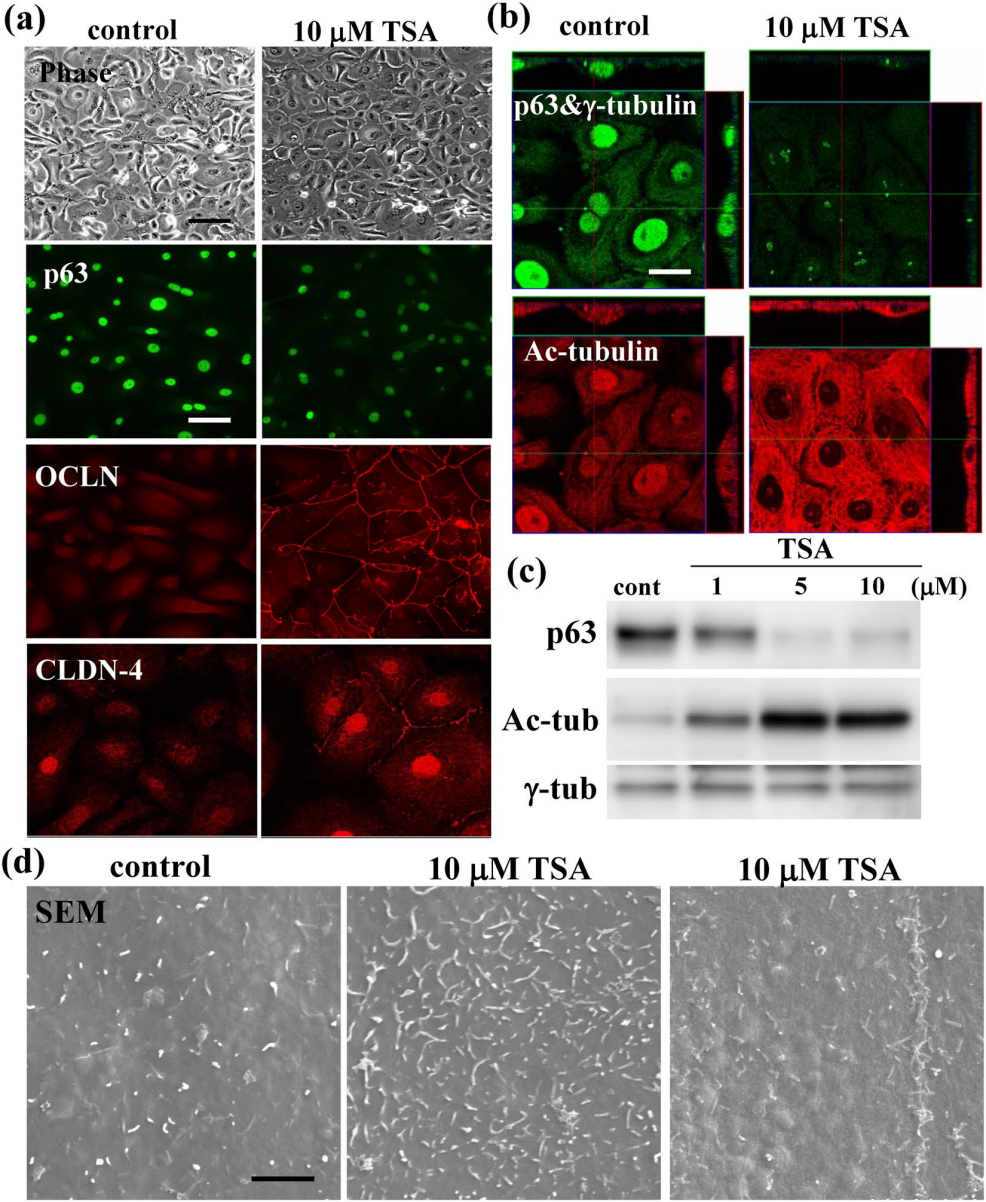
(**a**) Phase-contrast images and immunocytochemical staining for p63, OCLN and claudin-4 and (**b**) immunocytochemical staining for p63, γ-tubulin and Ac-tubulin in hTERT-transfected HNECs treated with 10 μM TSA. Bar: 20 μm. (**c**) Western blotting for p63, Ac-tublin and γ-tubulin in hTERT-transfected HNECs treated with 1, 5 or 10 μM TSA. Bar: 10 μm. (**d**) SEM in hTERT-transfected HNECs treated with 10 μM TSA. Bar: 3 μm.

To investigate whether the pan-inhibitor of HDACs Tricostatin A (TSA) affects tight junctions and microvilli, hTERT-HNECs cultured without FBS were treated with 1 and 10 μM TSA for 24 h. Treatment with TSA reduced p63 and induced CLDN-1 and -4 and Ac-tubulin in Westen blotting and enhanced the epithelial barrier function in a dose-dependent manner (Fig. 5b,c, Supplemental Fig. 4). The immunocytochemical results showed that OCLN and CLDN-4 presented at the membranes of the cells in which p63 was reduced by treatment with 10 μM TSA (Fig. 6a). Treatment with TSA was found to induce Ac-tubulin together with reduction of p63 by immunocytochemistry and Western blotting (Fig. 6b,c, Supplemental Fig. 5). Furthermore, in SEM analysis of 10 μM TSA-treated cells, the number of surface microvilli was increased compared to the control and tight sealed junction-like structures formed by the microvilli were also observed at the cell borders (Fig. 6d).

### Upregulation of tight junction proteins, barrier function and surface microvilli by the inhibitors of HDAC1 and HDAC6 in HNECs

**Figure 7:**
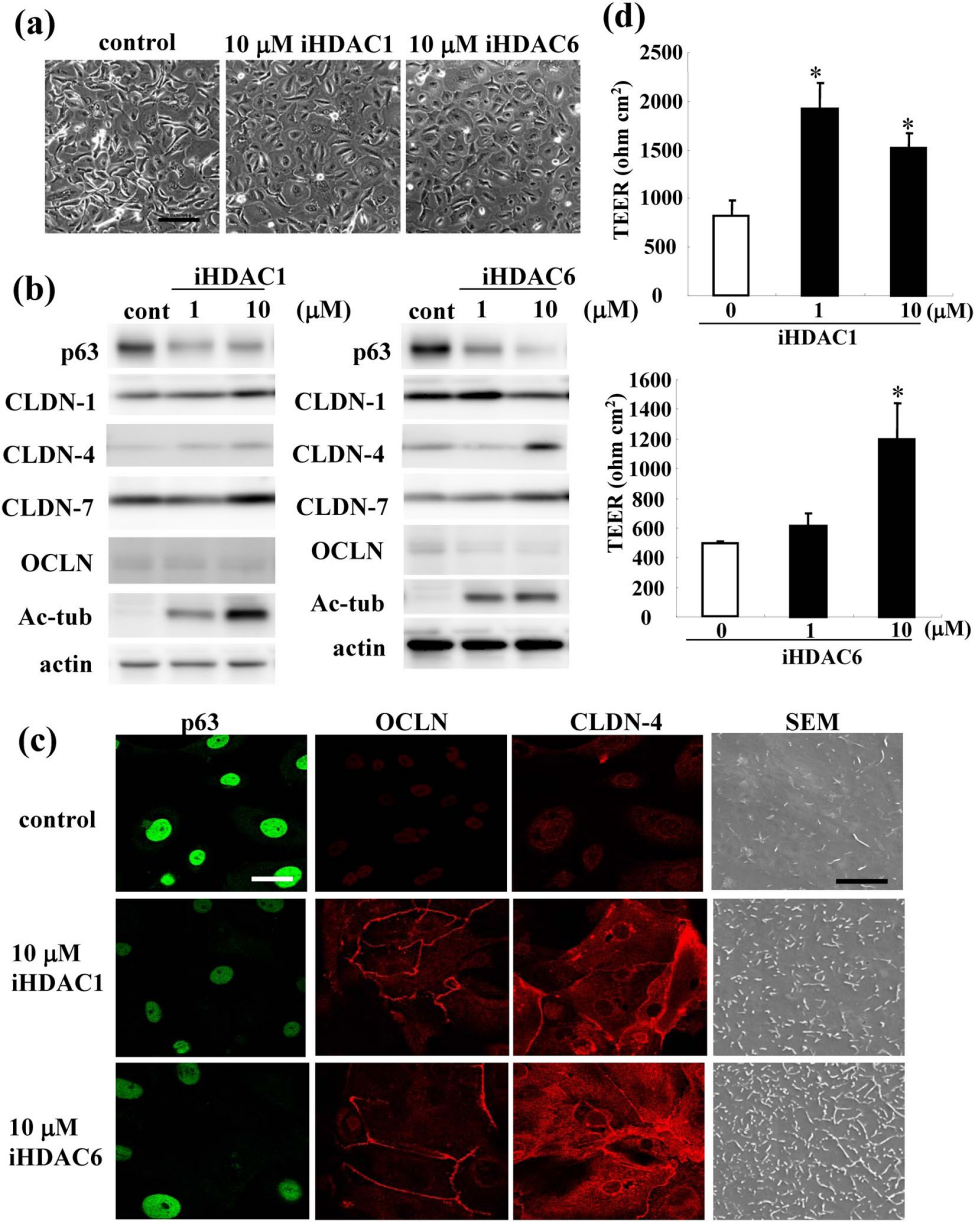
(**a**) Phase-contrast images of hTERT-transfected HNECs treated with 10 μM inhibitors of HDAC1 and HDAC6. Bar: 40 μm. (**b**) Western blotting for p63, claudin-1, -4, -7, OCLN and Ac-tublin in hTERT-transfected HNECs treated with 1 or 10 μM inhibitors of HDAC1 and HDAC6. (**c**) Immunocytochemical staining for p63, OCLN and claudin-4 and SEM in hTERT-transfected HNECs treated with 10 μM inhibitors of HDAC1 and HDAC6. White bar: 20 μm, black bar: 3 μm.

To investigate which specific HDAC inhibitors affected tight junctions and microvilli, hTERT-HNECs cultured without FBS were treated with inhibitors of HDAC1 and HDAC6 at 1 and 10 μM for 24 h. In Western blotting, treatment with inhibitors of HDAC1 and HDAC6 at 10 μM reduced expression of p63 and enhanced that of CLDN-4 and Ac-tubulin (Fig. 7b, Supplemental Fig. 6). Immunocytochemical results showed that OCLN and CLDN-4 presented at the membranes of the cells in which p63 was reduced by treatment with inhibitors of HDAC1 and HDAC6 at 10 μM (Fig. 7c). In SEM analysis of the cells treated with inhibitors of HDAC1 and HDAC6 at 10 μM, the number of surface microvilli was increased compared to the control (Fig. 7c).

### Downregulation of phospho-p38 MAPK by TAp63-siRNA, curcumin, TSA and HDAC1 inhibitor in HNECs

**Figure 8:**
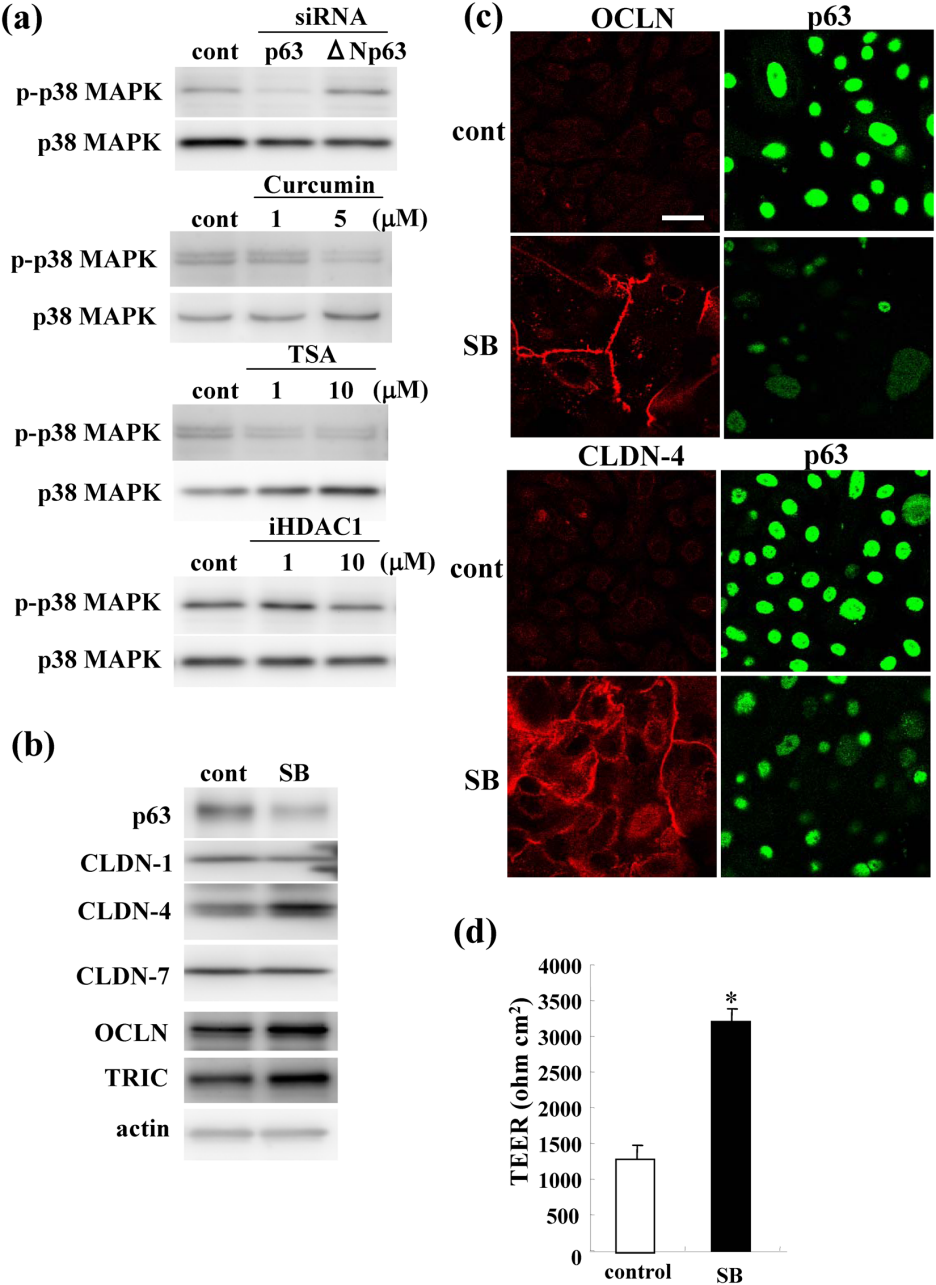
(**a**) Western blotting for phospho-p38MAPK and p38MAPK in hTERT-transfected HNECs transfected with siRNAs of p63 and ΔNp63 and treated with 1 or 5 μM curcumin, 1 or 10 μM TSA and 1 or 10 μM HDAC1 inhibitor. (**b**) Western blotting for p63, claudin-1, -4, -7, OCLN and TRIC, and (**c**) immunocytochemical staining for OCLN, claudin-4 and p63, and TEER values representing barrier function in hTERT-transfected HNECs treated with 10 μM p38MAPK inhibitor SB023580. Bar: 20 μm.

The epithelial barrier in HNECs is regulated via a distinct signal transduction pathway including p38 MAPK stress signaling^7, 8^. We investigated whether p38 MPAK signaling closely contributed to downregulation of p63 by treatment with siRNAs of TAp63 and ΔNp63, curcumin, TSA and an HDAC1 inhibitor in HNECs. When hTERT-HNECs without FBS were treated with TAp63-siRNA, curcumin, TSA and the HDAC1 inhibitor, phospho-p38 MAPK was douwnregulated by Western blotting (Fig. 8a, Supplemental Fig. 7A).

### Upregulation of tight junction proteins and barrier function via p63 by the p38 MAPK inhibitor SB230580 in HNECs

It is also known that p63 is regulated via p38 MPAK^40^. When hTERT-HNECs cultured without FBS were treated with the p38 MAPK inhibitor SB230580, it downregulated p63 and upregulated CLDN-4 in Western blotting (Fig. 8b, Supplemental Fig. 7B). Immunocytochemistry revealed that treatment with SB230580 led to the presentation of OCLN and CLDN-4 in the membranes and significantly induced TEER, i.e. barrier function (Fig. 8c,d).

## Discussion

In this study, we first found that downregulation of p63 regulated the epithelial barrier and ciliogenesis of the nasal epithelium in normal and diseased tissues. Inhibitors of HDACs, which were highly expressed in CRS and NPs, could induce the epithelial barrier and ciliogenesis via p63.

p63 regulates various cell–matrix and cell–cell adhesion complexes in the epidermis^23^. It contributes to the formation and maintenance of differentiated pseudostratified bronchial epithelium^24^ and regulates the target genes by direct interaction with Sp1^41^. CLDN-1 is a p63 direct target gene in epithelial development and p63 deficiency leads to inhibition of CLDN-1 in p63-null mouse keratinocytes^42^. CLDN-1 and CLDN-4 are in part controlled by the Sp1-containing critical promoter region^16^. In HNECs in the present study, knockdown of p63 by siRNAs of TAp63 and ΔNp63 induced expression of CLDN-1 and -4 with an increase of Sp1 activity and enhanced the barrier and fence functions. Furthermore, expression of the tricellular tight junction proteins TRIC and LSR was increased by the knockdown of p63. These results indicated that p63 negatively regulated the nasal epithelial TJ proteins and their functions in HNECs. In fact, in the nasal epithelium of CRS and NPs with an increase of p63-positive basal cells, disruption of epithelial TJs was observed.

TAp63 is a transcriptional target of NF-κB, which may play a role in cell proliferation, differentiation and survival upon NF-κB activation by various stimuli^43^. The regulatory feedback loop between TAp63 and NF-κB is involved in the activation of the cell-death process of cancer cells^44^. In HNECs, the NF-κB inhibitor curcumin inhibits NF-κB activity and upregulates CLDN-4 and OCLN^45^. Furthermore, RSV-infection also inhibits NF-κB activity and upregulates CLDN-4 and OCLN in airway epithelial cells^21, 29^. In the present study, treatment with curcumin and infection with RSV, downregulated p63 and upregulated CLDN-1 and CLDN-4, and CLDN-4 and OCLN, respectively, in HNECs. These findings suggested that the inhibition of NF-κB downregulated p63 expression and enhanced TJ proteins.

TJ proteins are upregulated in a kinase-dependent manner during cell differentiation induced by HDAC inhibitors^37^. The HDAC inhibitor TSA contributes to the activation of transcriptional factors p63 and Sp1^46^. IL-4-induced rat nasal epithelial barrier dysfunction can be blocked by TSA^47^. In the present study, HDAC1 was upregulated in the nasal epithelium of NPs, and HDAC6 was upregulated in sinusitis. In HNECs, treatment with TSA or specific inhibitors of HDAC1 and HDAC6 downregulated p63 expression and upregulated expression of some tight junction proteins, the barrier function and the numbers of surface microvilli. These results indicated that the HDAC inhibitors could protect against inhaled substances and pathogens through HNECs as a result of the barrier enhanced via p63.

The p38 MAPK/NF-κB signaling is involved in the epithelial barrier by means of various stimuli^48, 49^. The nasal epithelial barrier is regulated via a distinct signaling pathway including p38 MAPK^3^. p63 is in part regulated via p38 MPAK^40^. The NF-κB inhibitor curucumin and the HDAC inhibitor TSA affect the epithelial barrier via p38 MAPK/NF-κB^46, 50^. In the present study, the knockdown or downregulation of p63 by treatment with siRNAs of TAp63 and ΔNp63, curcumin, TSA and an HDAC1 inhibitor in HNECs inhibited the activity of phospho-p38MAPK. Conversely, the p38MAPK inhibitor SB23580 downregulated p63 expression and upregulated the epithelial barrier function with an increase of CLDN-4 expression. These results indicated that the bi-regulation between p63 and stress signal p38 MAPK/NF-κB was important in induction and maintenance of the nasal epithelial barrier.

Ciliary dysfunction is in part observed in chronic rhinosinsusitis^51, 52^. IL-6/STAT3 promotes the differentiation of ciliated cells from basal stem cells in airway epithelium^53^. Furthermore, in airway epithelial cells, a p63 (−) Myb (+) population derived from self-renewing p63 (+) Krt5 (+) epithelial progenitors, becomes ciliated cells under the influence of specific regulatory factors, including Notch and FOXJ1^31^. In hTERT-HNECs in the present study, the knockdown of p63 by siRNAs of TAp63 and ΔNp63, curcumin and the HDAC inhibitors, induced Ac-tubulin and enhanced the number of microvilli on the cell surface. Cilia-like structures were also observed in the some p63-knockdown cells. IL-6 expression was increased in p63-knockdown cells compared to the control in DNA array analysis (Table 1). All nuclei of control hTERT-HNECs were p63/CK5-positive (Fig. 2a). The p63/CK5-posive cells were markedly decreased by siRNAs of TAp63 and ΔNp63 in immunocytochemistry (Supplemental Fig. 2), whereas no change of CK5 expression in total cells was observed by Western blotting (Fig. 2b). These results suggested that in HNECs, the p63 (−) population might become ciliated cells via promotion by IL-6, although the effects of Notch and FOXJ1 remained unclear in the present study.

**Table 1:** List of gene probes which are up or down-regulated in hTERT-HNEC transfected with siRNA-p63.

Gene name	ID	Gene Bank ID	Fold-change
			control vs siRNA-p63
CLDN1	H200001413	NM-021101	1.4
CLDN4	H300004950	NM-001305	2.5
OCLN	opHsV0400004868	NM-002538	2.0
TJP3 (ZO-3)	H200002526	NM-014428	3.5
CGN (Cinglin)	H300009163	NM-028770	4.0
KRT7	H200003337	NM-005556	2.1
KRT5	H200001478	NM-000424	0.8
ELF3	H200007576	NM-004433	2.4
IL6	AHsV100021	NM-0006000.2	5.6

In conclusion, downregulation of p63 regulates the epithelial barrier and ciliogenesis of the nasal epithelium. After knockdown of p63 in primary bronchial epithelial cells, they do not proliferate and show marked senescence^24^. RSV infects the p63-positive basal cells of human bronchial epithelium and alters the epithelial differentiation^29^. Thus, it is possible that downregulation of p63 by various stimuli may alter the proliferation and differentiation of nasal epithelial cells.

## Electronic supplementary material

Supplemental Figs
