# Supplementary material for: The role of transcriptional factor p63 in regulation of epithelial barrier and ciliogenesis of human nasal epithelial cells
Source: Sci Rep. 2017 Sep 7;7:10935. doi: 10.1038/s41598-017-11481-w (PMC5589951; doi:10.1038/s41598-017-11481-w)
Supplement: Supplementary file 1 — Supplemental Figs [file 41598_2017_11481_MOESM1_ESM.pdf]

# The role of transcriptional factor p63 in regulation of epithelial barrier and ciliogenesis of human nasal epithelial cells

Yakuto Kaneko, Takayuki Kohno, Takuya Kakuki, Ken-ichi Takano, Noriko Ogasawara, Ryo Miyata, Shin Kikuchi, Takumi Konno, Tsuyoshi Ohkuni, Ryoto Yajima, Akito Kakiuchi, Shin-ichi Yokota, Tetsuo Himi, Takashi Kojima

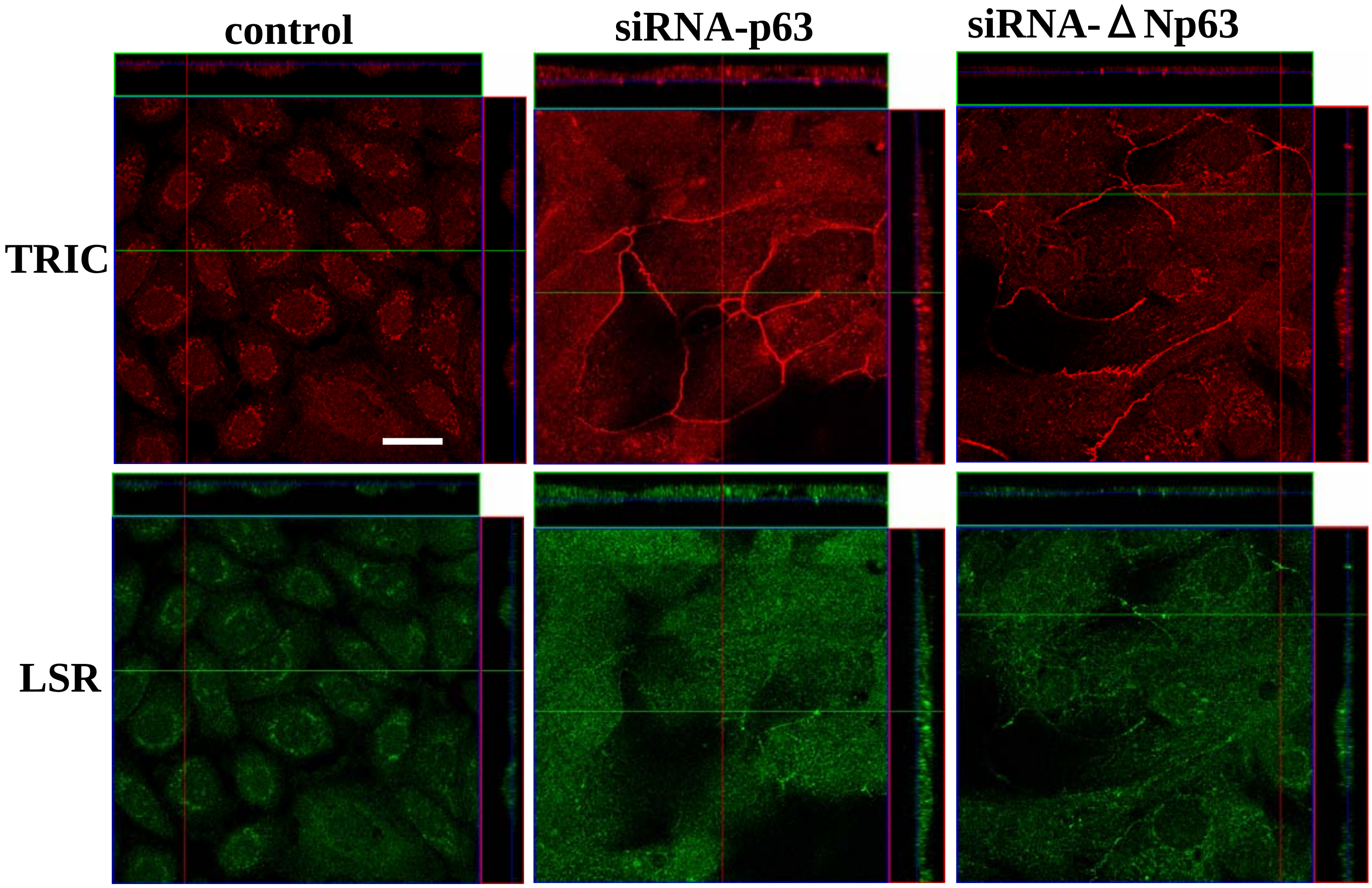


Supplemental Figure 1. Immunocytochemical staining for TRIC and LSR in hTERT-transfected HNECs transfected with siRNAs of p63 and ΔNp63. Bar: 20 μm.

Supplemental Figure 1

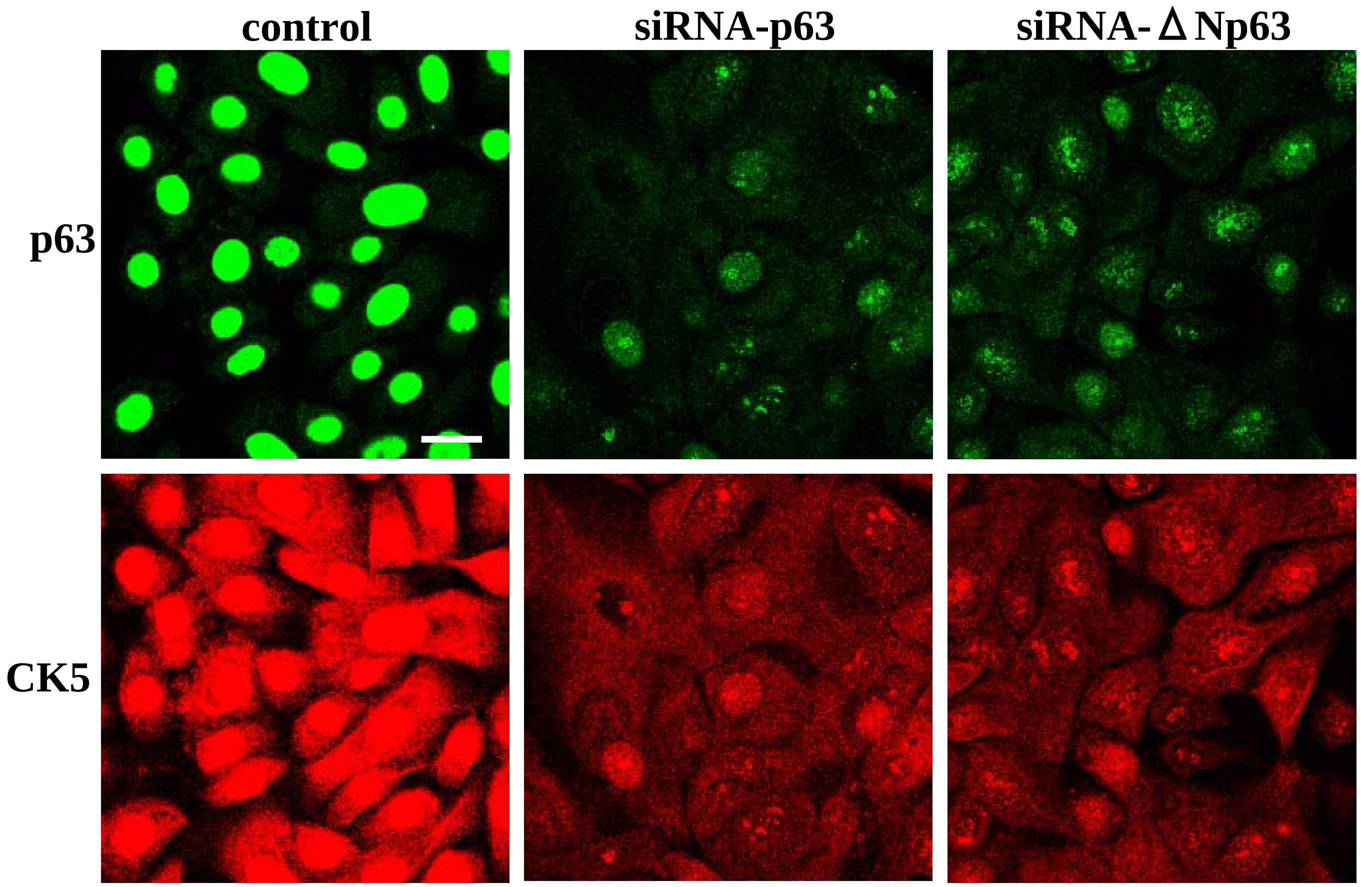


Supplemental Figure 2. Immunocytochemical staining for p63 and CK5 in hTERT-transfected HNECs transfected with siRNAs of p63 and ΔNp63. Bar: 20 μm.

**Supplemental Figure 2**

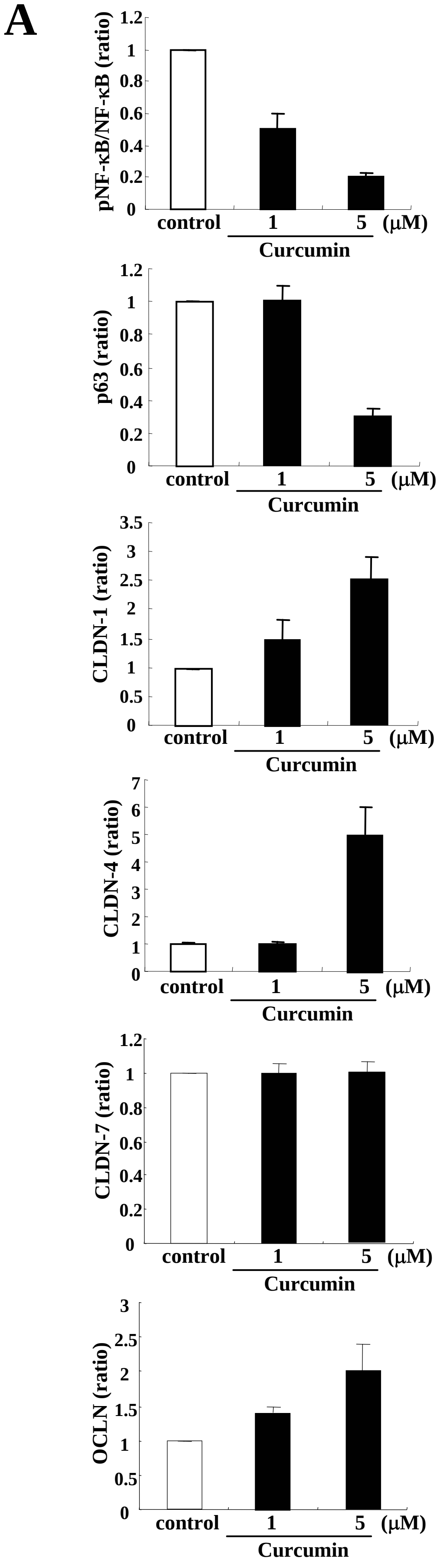


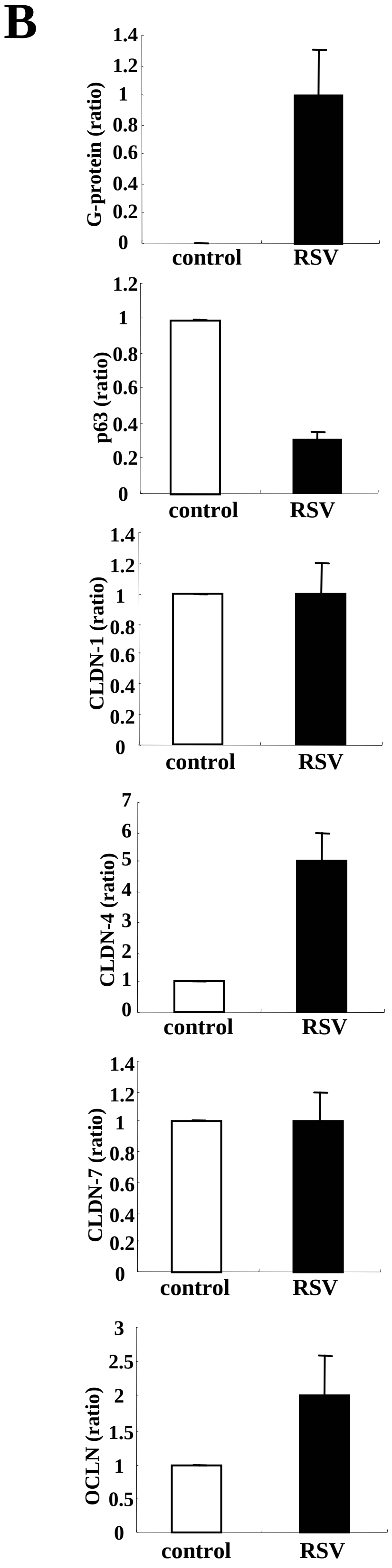


Supplemental Figure 3. (A, B) Bar graphs for the corresponding expression levels of Figure 4A and 4C.

**Supplemental Figure 3**

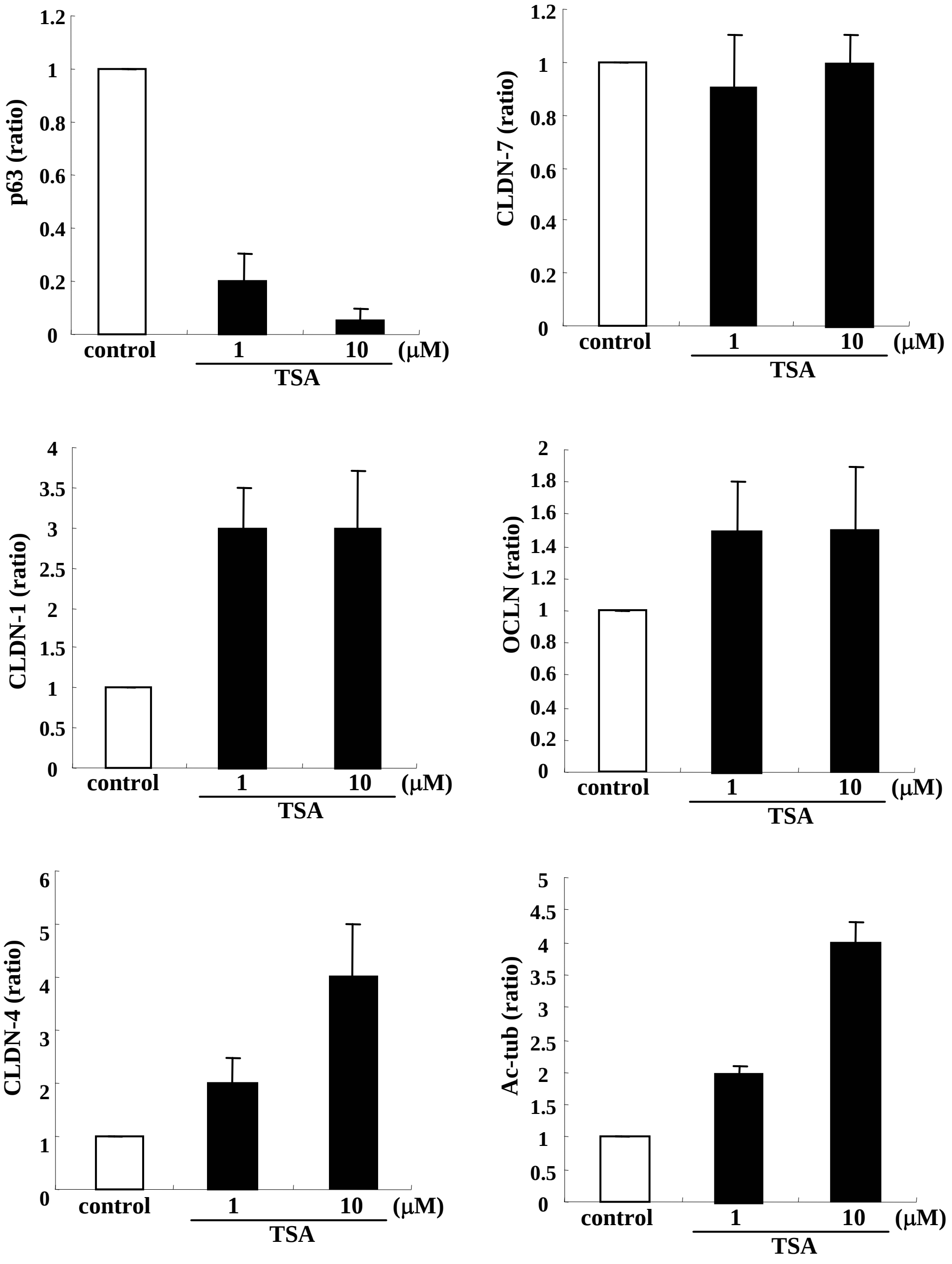


Supplemental Figure 4. Bar graphs for the corresponding expression levels of Figure 5B.

**Supplemental Figure 4**

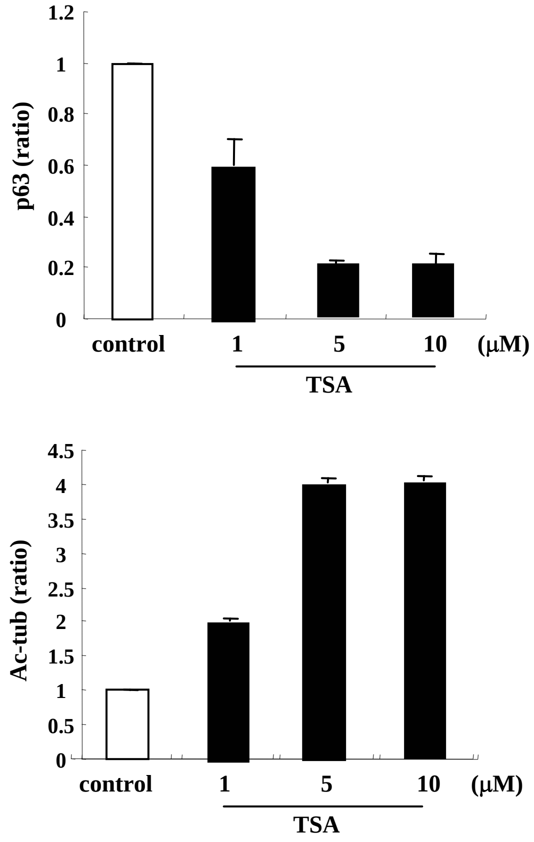


Supplemental Figure 5. Bar graphs for the corresponding expression levels of Figure 6C.

**Supplemental Figure 5**

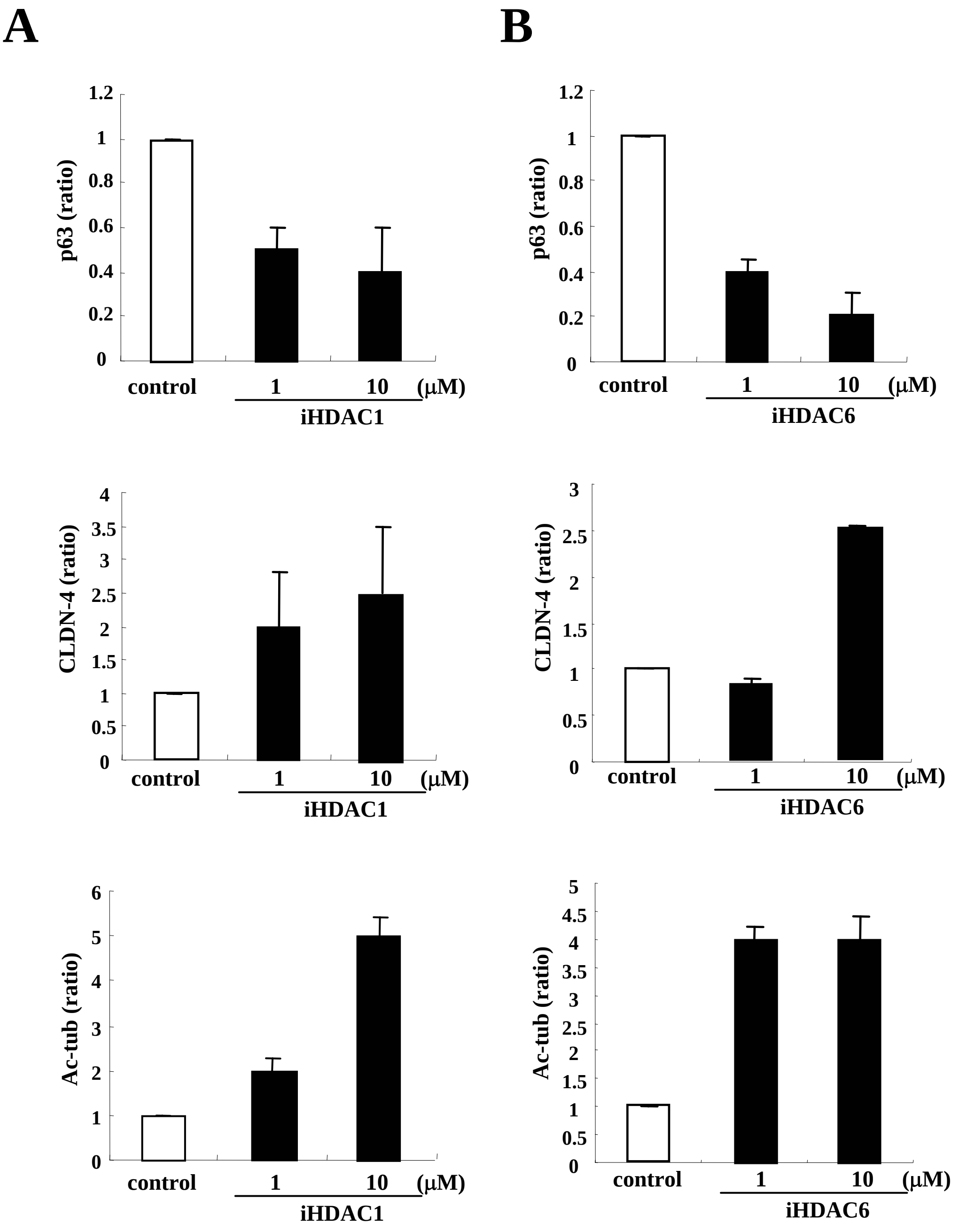


Supplemental Figure 6. (A, B) Bar graphs for the corresponding expression levels of Figure 7B.

**Supplemental Figure 6**

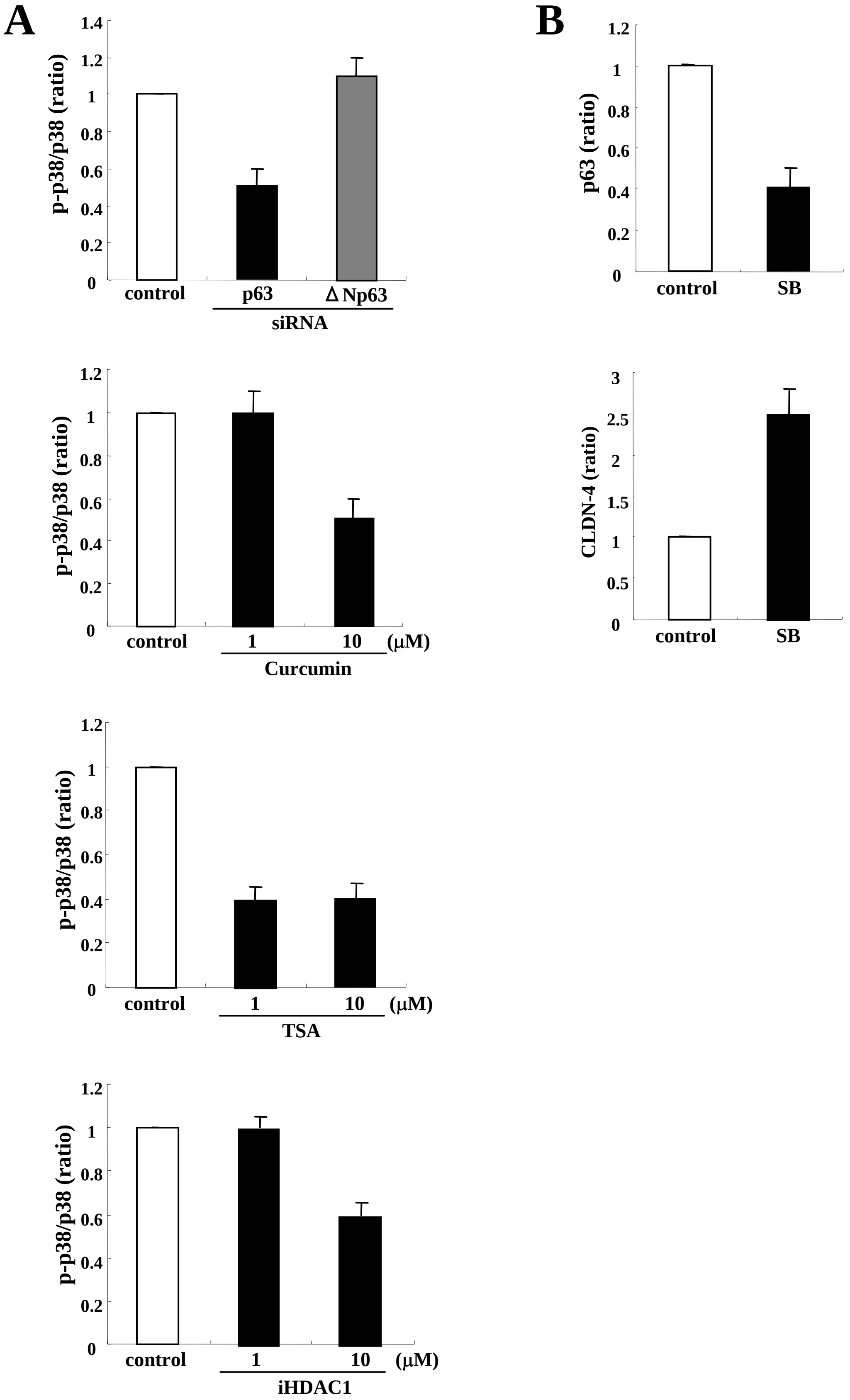


Supplemental Figure 7. (A, B) Bar graphs for the corresponding expression levels of Figure 8A and 8B.

**Supplemental Figure 7**
